# Comparative Analysis of Aligned Conductive Electrospun Mats of PLGA-Polypyrrole for Neural Tissue Engineering: Encapsulation vs Coating

**DOI:** 10.1021/acsomega.4c11594

**Published:** 2025-05-24

**Authors:** Vanessa Oliveira Castro, Sebastien Livi, Laura Elena Sperling, Marcelo Garrido dos Santos, Katja Heise, Claudia Merlini

**Affiliations:** † Mechanical Engineering Department, Universidade Federal de Santa Catarina (UFSC), 88040-900 Florianópolis, Brazil; ‡ Ingénierie des Matériaux Polymères, Universite Claude Bernard Lyon 1, INSA Lyon, Université Jean Monnet, CNRS UMR 5223, F-69621 Villeurbanne Cédex, France; § Hematology and Stem Cell Laboratory, Faculty of Pharmacy, Universidade Federal do Rio Grande do Sul (UFRGS), 90010-150 Porto Alegre, Rio Grande do Sul, Brazil; ∥ Materials Engineering Special Coordination, Universidade Federal de Santa Catarina (UFSC), 89036-256 Blumenau, Santa Catarina, Brazil; ⊥ Institute of Nanoscale and Biobased Materials, Faculty of Materials Science and Materials Technology, 26545 Technische Universität Bergakademie Freiberg, 09599 Freiberg, Germany

## Abstract

The electrical conductivity of aligned electrospun mats has shown promising results in neural tissue engineering due to its capacity to stimulate neural cell growth and cell differentiation. Herein, we compare two processing routes of conductive aligned electrospun mats to be used as a conduit in nerve regeneration. In the first route, polypyrrole (PPy) nanoparticles were encapsulated into poly lactic-*co*-glycolic acid (PLGA) fibers. In the second route, PLGA fibers were coated with PPy by in situ oxidative polymerization of pyrrole. Their microstructure, physicochemical, electrical, and biological properties were investigated and compared. The results showed that both routes achieved well aligned fiber structures, without bead defects and fiber diameters between 500 nm and 1 μm (after the coating). Regarding the first route, PPy-encapsulated fibers increased electrical conductivity by one magnitude order compared to neat PLGA fibers. This result derives from the shielding encapsulation effect caused by the polymer matrix. Nevertheless, PPy-encapsulated fibers presented higher mechanical properties and biocompatibility, compared to PPy-coated fibers. In the second route, PPy-coated fibers presented significantly higher responses in terms of wettability and electrical properties. However, these PPy-coated mats exhibited high cytotoxicity levels as evidenced by MTT assay, and proved to be difficult to roll into a conduit. In conclusion, the first route was considered more suitable for further studies, making them a promising material for the development of aligned electrospun fibers conduits for nerve regeneration.

## Graphical abstract

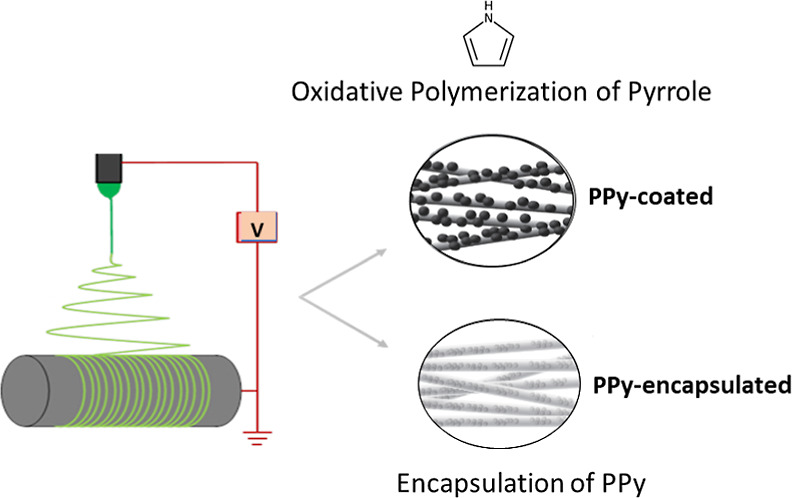

## 1 Introduction

In the field of neural tissue regeneration, the standard approach for restoring nerve function in cases of severe nerve injury is the use of nerve autografts or nerve conduits. Nowadays, nerve autografts are still considered the gold standard technique.^1^ A nerve autograft involves repairing an injured nerve using a nerve graft from another part of the patient’s body. Nevertheless, there are several limitations to this technique, including the limited availability of donor sites, size mismatch between donor and recipient nerves, and also the loss of nerve function at the donor site.^2^ To overcome these drawbacks, nerve conduits have been developed as an alternative strategy.^3–5^ Several nerve conduits are already commercially for clinical use, such as neuragen (type 1 collagen), neurotube (polyglycolic acid), and neurolac (poly­(dl-lactide-ε-caprolactone)).^6^ Although these conduits provide the basic needs of structural support, cell barrier function, and biocompatibility with degradability, functional recovery is limited when compared to nerve autografts.^7^

Several techniques have been reported in the literature for producing a new generation of nerve conduits, including freeze-drying, dip coating, and electrospinning.^8–11^ This new generation of nerve conduits aims to overcome the limitations of the previous generations, which were mainly associated with nonbiodegradable, rigid structures, and lack of biomimetic features. Among these, electrospinning has shown great potential, primarily due to the 3D microstructure composed of micro- and/or nanofibers, which provides a high surface area for cell proliferation. Additionally, electrospun conduits developed through electrospinning offer deformability and mechanical performance required to withstand physiological activities.^12^ The method also provides the possibility to obtain aligned fibers with a rotating collector. Nerve conduits made from aligned electrospun mats have demonstrated an increase in neural cell spreading along the electrospun fiber axis. Zhou et al. reported that neurite lengths increased by approximately 120%—from 25 to 55 μm, when seeded on aligned PPy-coated electrospun fibers. These values correspond to the control group in 48 h PC12 cell culture, without any additional stimulation.^13^ Physically guiding the linear growth of axons through an injury site provides structural support for cell growth through the microarchitecture of the fibers. Additionally, this microarchitecture has the potential to preserve the native organization of regenerating axons throughout the lesion site and into the adjacent host tissue, increasing the probability of achieving a higher functional nerve recovery.^14^

To stimulate nerve regeneration, the strategy of adding electrical properties to electrospun mats has become notable in nerve regeneration research.^15,16^ Neural cells are sensitive to electrical stimulation, and are able to sense and to be stimulated by the propagation of electrical signals through the electrically conductive fibers.^17^ Recent studies have also explored the potential of graphene-based nerve conduits.^18–20^ However, concerns have been raised regarding the toxicity at low concentrations, and also long-term biocompatibility. For instance, researchers have found that long-term exposure to low doses of graphene oxide nanoparticles in vivo (mice) led to accumulation in the reproductive organs and reproductive toxicity. They also resulted in neurobehavioral toxicity, indicated by reduced locomotion, which shortened the lifespan of the animals.^21,22^ Conductive polymers, such as polypyrrole (PPy), have attracted significant attention as a promising biomaterial for nerve regeneration due to their biocompatibility, and chemically suitable surface for cell adhesion.^23,24^

Some studies have demonstrated that in electrically excitable cells, such as neural cells, conductive polymers significantly enhance neurite outgrowth and cell spreading.^25,26^ In some cases, this effect occurs without any additional external electrical stimulation, while in others, the regeneration can be enhanced by external stimulation. There is no consensus in the literature as to whether external electrical stimulation is necessary for neural regeneration. Its application can represent an additional challenge for the patient’s body, for instance the insertion of electrodes. Thus, the need for external stimulation may add complexity to the process more complex without providing significant benefits.

Two main routes are commonly used to obtain electrospun conductive conduits: (i) encapsulation of PPy by adding conductive nanoparticles in the polymer solution followed by electrospinning,^27^ or (ii) coating the electrospun fibers with conductive.^4,10,13,23^ Coating the electrospun fibers with conductive nanoparticles, mainly PPy has been shown in more recurrent studies with good results in terms of electrical conductivity and biological responses. There are different techniques for the coating of the fibers, such as in situ oxidative polymerization, admicellar polymerization, and vapor phase polymerization.^10,15,28^ All these routes result in different coating deposition uniformity, conductivity, and biological responses.

The present study was based on previous studies by our research group.^29–31^ The encapsulation route was based on the approach of our group, which tested the influence of different mass fractions of PPy in thermoplastic polyurethane (TPU) electrospun mats to investigate their properties.^30^ An optimal concentration of 7.5 wt % PPy in the electrospun mats presented a higher biological response; however, concentrations above this level were considered cytotoxic. At this optimal PPy concentration, an electrical conductivity of 3.7 × 10^–12^ S·cm^–1^ was obtained. A minimum electrical conductivity value to achieve biological responses has not been defined in the literature. However, good results in terms of functional recovery were found, being 0.1 S·cm^–1^ for coated electrospun mats and 10^–5^ S·cm^–1^ for electrospun mats containing PPy.^5,23^ Further studies are required for a better comparison. The coating route used was the in situ oxidative polymerization of pyrrole, based on the work of Merlini et al. (2014). They developed highly sensitive PPy-coated electrospun mats with an electrical conductivity value of 1.6 S·cm^–1^.^29^ No biological assays were tested in this study.

Herein, we present the development of aligned electrospun mats prepared by two different routes: (i) PPy-encapsulated fibers by adding 7.5 wt % of PPy nanoparticles to PLGA solution followed by electrospinning and (ii) PPy-coated fibers, by coating PLGA fibers with PPy by in situ polymerization. This study investigated the effects of these two processing routes on the microstructure, thermal and mechanical properties, physicochemical characteristics, electrical performance, and cytocompatibility of electrospun mats, with a focus on their potential use in nerve regeneration.

## 2 Experimental Section

### 2.1 Materials

PLGA (l-lactide and glycolide 82:18) (Resomer LG 824 S), with an inherent viscosity of 1,7–2,6 [dL/g], was purchased from Evonik Industries (Germany). Dimethylformamide (DMF) and dichloroethane (DCE) were purchased from Vetec Química Fina Ltda and used as received. Pyrrole monomer, 98%, was purchased from Sigma-Aldrich (Germany). Dodecylbenzenesulfonic acid (DBSA) (Aldrich) and Iron­(III) chloride hexahydrate, FeCl_3_·6H_2_O, analytical grade (Vetec), were used as received.

### 2.2 Synthesis of Polypyrrole Nanoparticles

The synthesis of polypyrrole (PPy) nanoparticles was performed through in situ oxidative polymerization, following the procedure described by Ramoa et al.^32^ Initially, the DBSA anionic surfactant (1.88 g) was dispersed in 0.05 L of distilled water in a 250 mL beaker, then 2 g (0.3 mol/L) of pyrrole (Py) was added under stirring. After 10 min, a solution of 16.2 g of FeCl_3_·6H_2_O in 0.05 L distilled water was added dropwise to the aqueous dispersion containing pyrrole and DBSA. The oxidant/monomer and monomer/surfactant molar ratios used were 2/1 and 5/1 mol/mol, respectively. Polymerization was maintained by magnetic stirring for 1 h at a temperature of 21 ± 2 °C. After 24 h (without stirring), the PPy·DBSA (named PPy) was filtered and washed several times with distilled water to extract residues and byproducts from the reaction. After washing, the PPy was dried in a vacuum oven at 60 °C, until its mass remained constant. After drying, the particles tend to agglomerate and to facilitate the separation of the agglomerates, as the last step, the PPy was sieved using a mesh sieve mortar with an opening of 75 mm/μm (200 mesh, brand Bertel) and stored at room temperature.

### 2.3 Electrospinning

PLGA neat and PLGA containing PPy nanoparticle solutions were prepared for the electrospinning. PLGA was solubilized in DMF/DCE 3:1 v/v at 50 °C for 5 h, resulting in a solution of 7.4 wt % PLGA, as previously described.^31,33^ PPy nanoparticles, with a mass fraction of 7.5 wt % (based on the weight of PLGA), were added to the PLGA solution under magnetic stirring for 15 min, named PPy-encapsulated. After that, the solution was sonicated with a tip ultrasonic probe (400 W and 60 Hz), for 5 min. This particular mass fraction was selected based on a previous study wherein it was determined to be the maximum concentration used without cytotoxic response.^30^ The suspensions were electrospun through a 5 mL syringe, with a needle with an internal diameter of 0.6 mm, coupled to a syringe pump (Instor Apparatus). The rotating metallic collector, with diameter of 5.5 cm, was wrapped with an aluminum sheet and grounded, while the positive pole was connected to the syringe. The power supply used to generate the electric field has a direct current of up to 30 kV (Instor Apparatus). The PLGA neat and PPy-encapsulated solution was electrospun at an electrical potential of 18 kV, a distance between needle and collector of 25 cm, a flow rate of 1 mL/h, and a speed of 2500 rpm (7.2 m/s).

### 2.4 PPy-Coating Process

PLGA-aligned mats were coated with PPy by in situ oxidative polymerization of pyrrole, using FeCl_3_·6H_2_O as an oxidant agent. Pyrrole monomer was distilled under vacuum before use. The in situ oxidative polymerization was performed according to the study carried out by Merlini et al. (2014).^29^ First, two solutions were prepared: pyrrole (0.1675 g) in 25 mL of distilled water (solution A), and the oxidant agent FeCl_3_·6H_2_O (1.35 g) in 25 mL of distilled water (solution B), both under magnetic stirring for 10 min. The oxidant/monomer molar ratio was 2/1 mol/mol and the concentration of PPy was 0.05 mol·L^–1^. Mats with an area of 30 mm^2^ were immersed in solution B. Then, solution A was dripped into solution B with the presence of the mats. Polymerization was carried out over 5 h under mild magnetic agitation. After this time, PPy-coated mats were washed three times with distilled water and dried in a desiccator.

### 2.5 Preparation of the Electrospun Nerve Conduits

For the development of a prototype of an electrospun conduit, an electrospun mat with 14 mm × 40 mm was cut and rolled up using a metal stick with a diameter of 1.5 mm, aiming at a nerve gap of 10 mm. Thereby, a final conduit with 1.7 mm outer diameter and 14 mm length was produced, as illustrated in Figure 1.

**Figure 1:**
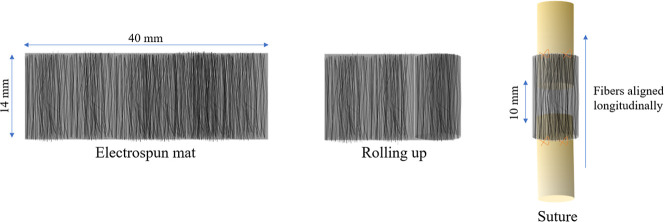
Electrospun conduit development process.

### 2.6 Material Characterization

#### 2.6.1 Surface Morphology and Physicochemical Properties

Aligned fibrous mat micrographs were obtained using a scanning electron microscope (SEM) (Jeol JSM-6390LV). All samples were sputtered with gold and analyzed under an accelerating voltage of 10 kV. From the SEM images, the fiber diameter was obtained using image analysis software (ImageJ, National Institutes of Health, Bethesda, USA).

The morphology of the PPy nanoparticles was analyzed by transmission electron microscopy (TEM) (Jeol JEM-1011) with a voltage acceleration of 80–100 kV. The nanoparticles were deposited on a 300-mesh copper mesh grid with carbon film.

The surface wettability of the aligned electrospun mats was characterized by the sessile drop method using a goniometer (KRÜSS Drop Shape Analyzer DSA25, USA). A water droplet of 5 μL was dropped from a syringe perpendicular to the sample surface. After the water was applied on the surface for 5 s, the droplet arc and the contact angle at the interface were traced and recorded. The contact angle was measured five times from different positions and an average value was calculated.

#### 2.6.2 Spectroscopic Analysis

Attenuated total reflectance Fourier transform infrared (ATR-IR) spectroscopy was performed on a Bruker spectrometer (model TENSOR 27). Spectra were recorded from 4000 to 400 cm^–1^, by accumulation of 32 scans, and with a resolution of 4 cm^–1^.

X-ray photoelectron spectroscopy (XPS) analysis was conducted to characterize the elemental composition of the fiber surfaces. The analyses were performed with an ESCALAB 200Xi spectrometer with an Al–Kα radiation source. The high-resolution spectra were fitted with a series of Gaussian peaks.

#### 2.6.3 Mechanical Analysis

The mechanical performance was characterized by tensile testing on an MTS 2/M electromechanical testing machine at room temperature (22 ± 1 °C) and at a relative humidity of 30 ± 5%. The electrospun mats were cut to dimensions of 10 mm × 30 mm, with a thickness of approximately 0.05 mm. The mechanical tests were performed with a constant crosshead displacement of 5 mm.min^–1^. DMA experiments were carried out on a dynamic mechanical analyzer DMA/SDTA861E, (Mettler Toledo) in tensile mode on rectangular specimens with a width of a maximum width of 3 mm and a length of 9 mm. The analyses were performed at a frequency of 1 Hz, maximum strain of 0.67%, scanning the temperature range (from 25 to 110 °C), at a heating rate of 5 °C·min^–1^.

#### 2.6.4 Electrical Conductivity

The electrical conductivity of the mats was measured by the four-probe standard method using a Keithley 6220 current source to apply the current and a Keithley model 2400 electrometer. The electrical conductivity (σ) (S·cm^–1^) was determined by eq 1, where, *I* is the electrical current (A), *V* is the electrical potential difference (V), w is the sample thickness (cm), and (ln 2)/π is a correction factor.

1 $$\sigma =\frac{I}{V}\times \frac{\ln2}{\pi w}$$

#### 2.6.5 Cytocompatibility

Biomaterial cytotoxicity was assessed using PC12 cells by MTT [3-(4,5-dimethylthiazol-2-yl)-2,5-diphenyltetrazolium bromide] cell viability assay. PC12 cells were cultivated on top of the electrospun mats and MTT cell viability was performed. The mats were cut into circle-like shapes, sterilized by UV light for 30 min, and placed at the bottom of a 48-well culture plate, and seeded with 5000 PC12 cells. PC12 cells (ATCC CRL-1721), are an established neural cell model derived from a pheochromocytoma of the rat adrenal medulla. The cells were permeabilized with 0.1% Triton X-100 in PBS for 5 min at room temperature. This cell line was cultured in culture flasks with DMEM high medium supplemented with 10% fetal bovine serum inactivated by heat, 5% horse serum and 1% penicillin/streptomycin and maintained at 37 °C in an incubator with a humidified atmosphere with 5% CO_2_. All assays were performed in triplicate.

The cells were cultured on the mats for 1, 3, or 7 days at 37 °C and 5% of CO_2_ and the cell viability was analyzed on the 3 times frames according to the MTT standard protocol. Briefly, the cell culture medium was removed from the well and replaced with 400 μL of 5 mg/mL MTT solution in PBS and incubated for 2 h at 37 °C and 5% CO_2_. Afterward, the MTT solution was replaced by dimethyl sulfoxide (DMSO) to completely lyse cell membranes and solubilize crystals produced by MTT metabolism. After solubilization, 200 μL of this solution was transferred to a 96-well plate and the absorbance was measured using a spectrophotometer at wavelengths of 540 and 620 nm.

#### 2.6.6 Rhodamine Phalloidin/DAPI Staining

PC12 cells were fixed with 4% paraformaldehyde for 20 min at room temperature on day 7 in culture. The actin cytoskeleton was stained with 1:100 phalloidin/rhodamine (Sigma-Aldrich) for 40 min. Cell nuclei were stained with (4′,6-diamidino-2-phenylindole) DAPI (Life Technologies) for 5 min. Following this, images were taken using a Zeiss fluorescence microscope.

#### 2.6.7 Statistical Analysis

Statistical analysis was performed using GraphPad Prism 9 applying one-way analysis of variance (ANOVA), followed by the Tukeýs post hoc test. A *p* value ≤0.05 was considered statistically significant. Each group of experiments was performed at least three times, and the results were expressed as mean ± standard deviation.

## 3 Results and Discussion

### 3.1 Surface Morphology and Physicochemical Properties

PLGA, PPy-encapsulated, and PPy-coated electrospun mats micrographs are presented in Figure 2. The electrospun mats of neat PLGA are composed of aligned fibers with a diameter of 561 ± 149 nm. From the first route, the incorporation of PPy nanoparticles (diameter of 95.1 ± 24.7 nm) led to the maintenance of the fiber alignment combined with a slight reduction of the fiber diameter to 495 ± 191 nm. This behavior can be related to the increase in the ionic conductivity of the solution, which increases the stretching of the fibers during electrospinning. While, from the second route after coating, the PPy-coated fibers maintained the fiber alignment, and increased their diameter to 908 ± 219 nm, due to the deposition of PPy on the fibers’ surface. In addition, we can observe on the PLGA fiber surfaces the formation of a continuous PPy layer with the presence of a few agglomerates.

**Figure 2:**
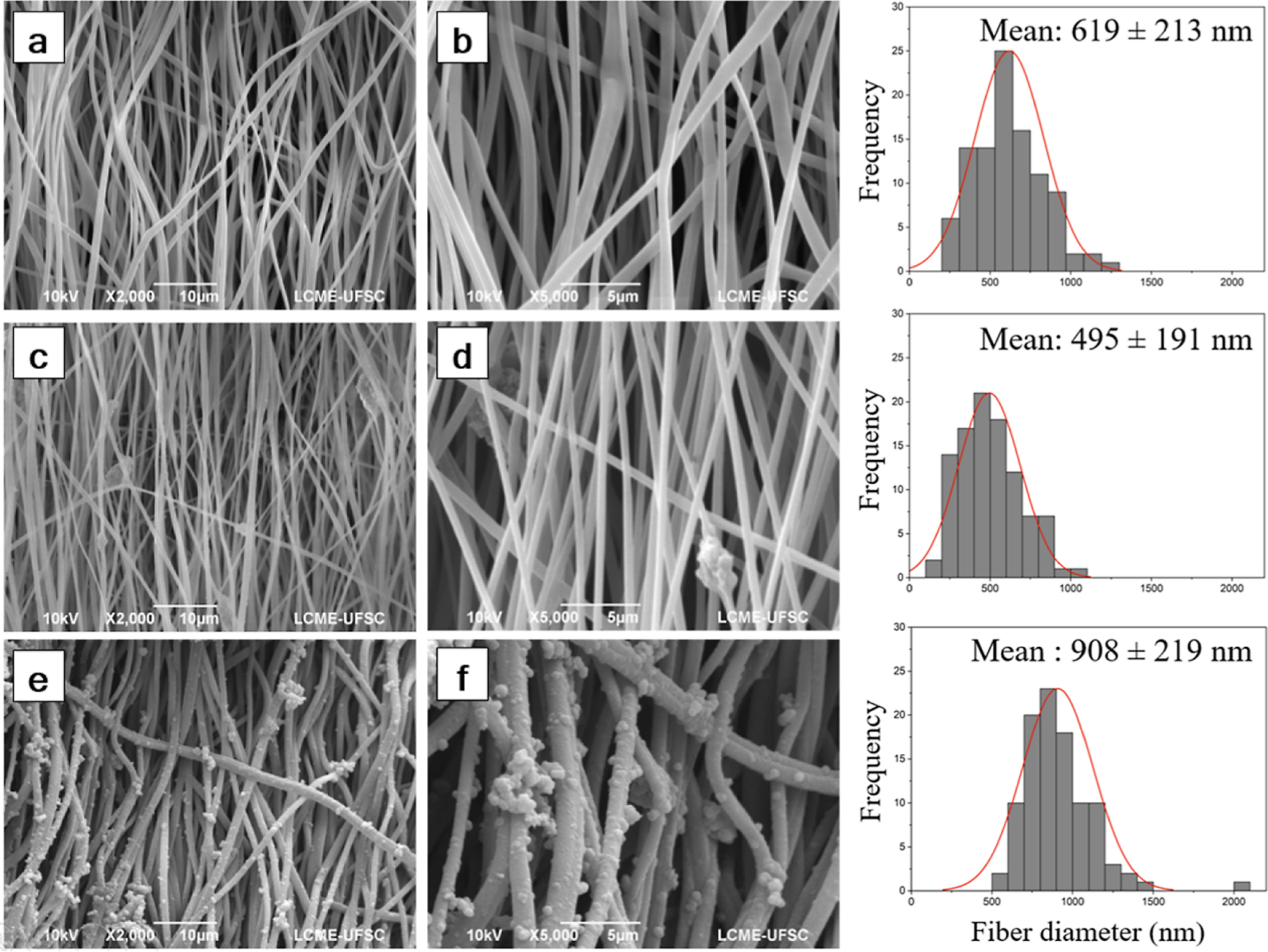
SEM images of (a,b) PLGA, (c,d) PPy-encapsulated, and (e,f) PPy-coated electrospun mats. Magnification of 2000× and 5000×.

To visualize the PPy nanoparticles morphology and also to visualize the distribution of the PPy inside the fibers obtained from the first route, TEM was performed and is presented in Figure 3. It is important to highlight that due to the large fiber diameter after coating with PPy, TEM analysis of PPy-coated could not be performed. As can be seen in Figure 3a, PPy nanoparticles exhibit a roughly spherical shape, with an average size of around 100 nm and a high surface area, which can promote their agglomeration within the fibers. Figure 3b shows the encapsulation of the PPy nanoparticles within the PLGA matrix. A potential strategy to address this issue could be to increase the ultrasonication time, and/or incorporate ionic liquids to improve filler dispersion in the polymer matrix. Ionic liquids are well-known for improving particles dispersion by reducing the interactions between particles, and also stabilizing them through their strong ionic interactions and physicochemical properties.^34,35^

**Figure 3:**
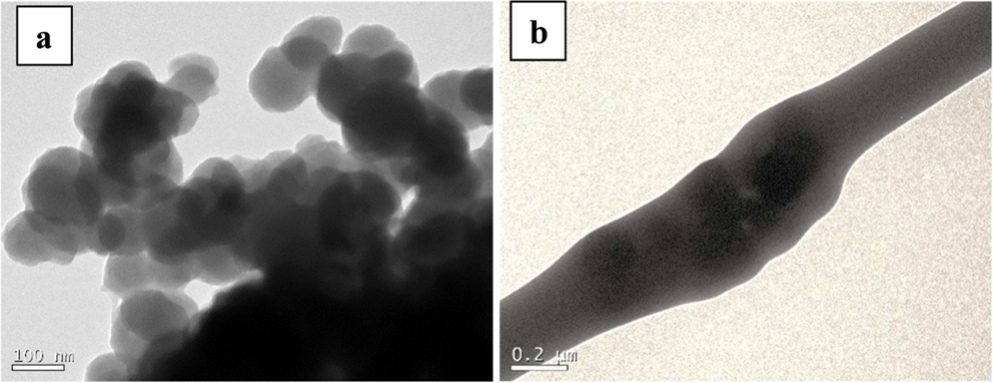
TEM images of (a) PPy nanoparticles and (b) a single PPy-encapsulated electrospun fiber.

Hydrophilicity is a key parameter of biomedical materials, due to its strong influence on cell activities, such as cell adhesion, growth, proliferation, and migration.^36^ Figure 4 presents the water contact angles of the PLGA, PPy-encapsulated, and PPy-coated mats. The value obtained for the pure PLGA mat was 148° ± 3, indicating an ultrahydrophobicity surface, which could be a topographical effect (like lotus leaf). According to the literature, polyesters such as PLA and PLGA, are well-known hydrophobic polymers.^37,38^ The intrinsic hydrophobic nature of PLGA is a consequence of the presence of methyl side groups from the acid lactic monomer. Another variable, that may influence the water contact angle, is the surface topography. Aligned fibers offer less contact between the fiber mat surface and the water droplet, when compared to random fibers, implying higher hydrophobicity. As can be seen in a study by Ershuai et al. (2016), the water contact angle of a PLGA mat comprised of random fibers was 112° ± 0.4,^39^ which is less hydrophobic when compared with the presented study. For PPy-encapsulated electrospun mats, there was a slight decrease to 139° ± 3, but the mats were still present high hydrophobic. PPy-coated mats became hydrophilic with a water contact angle of 82° ± 7. This decrease was expected and can be explained by the presence of PPy on the fiber surface. PPy presents a lone pair of valence electrons in the nitrogen atom, which may induce hydrogen bonding between the heterocyclic pyrrole and water molecules, providing the PPy hydrophilicity.^40^

**Figure 4:**
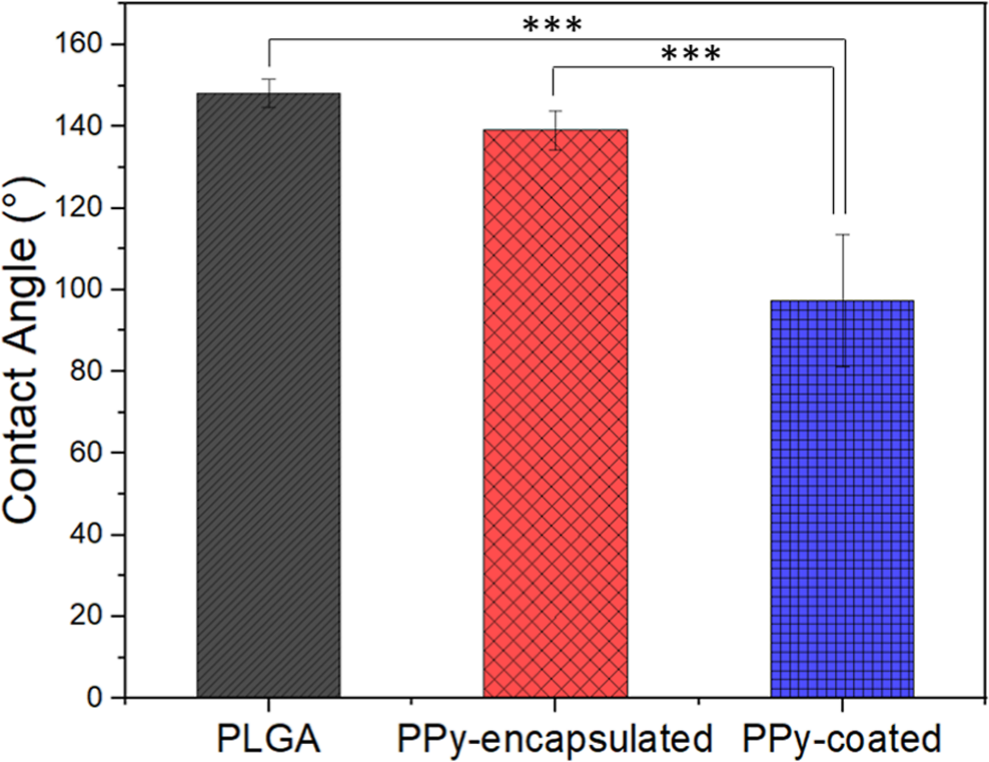
Average water contact angles of PLGA, PPy-encapsulated, and PPy-coated. *P < 0.05, **P < 0.01 and ***P < 0.001 (*n* = 5).

XPS analysis was conducted to determine the surface chemical composition of the electrospun mats, and also to evaluate the coating efficiency. As shown in Figure 5a, the XPS spectrum presents the elemental composition of the PLGA mat. Two peaks were detected, corresponding to the elements C 1s and O 1s, as expected. Figure 5b, related to PPy-encapsulated presented a C 1s at 287.48 eV (C–N). This peak C–N is may be attributed to the detection of PPy near fiber surface. The absence of the nitrogen peak is attributed to the encapsulation of PPy within the fiber. Furthermore, the depth penetration of XPS is between 1 and 10 nm, which makes the detection of nitrogen difficult. Figure 5c, nitrogen (N) atoms were detected on the surfaces of the samples, as expected, due to the PPy coating. The peak at 401.71 eV (O–N) indicates the PPy was successfully anchored on the PLGA surface. Chlorine peak was also detected around 200 eV, indicating the presence of residual iron chloride (FeCl_3_·6H_2_O) from the in situ oxidative polymerization of pyrrole.

**Figure 5:**
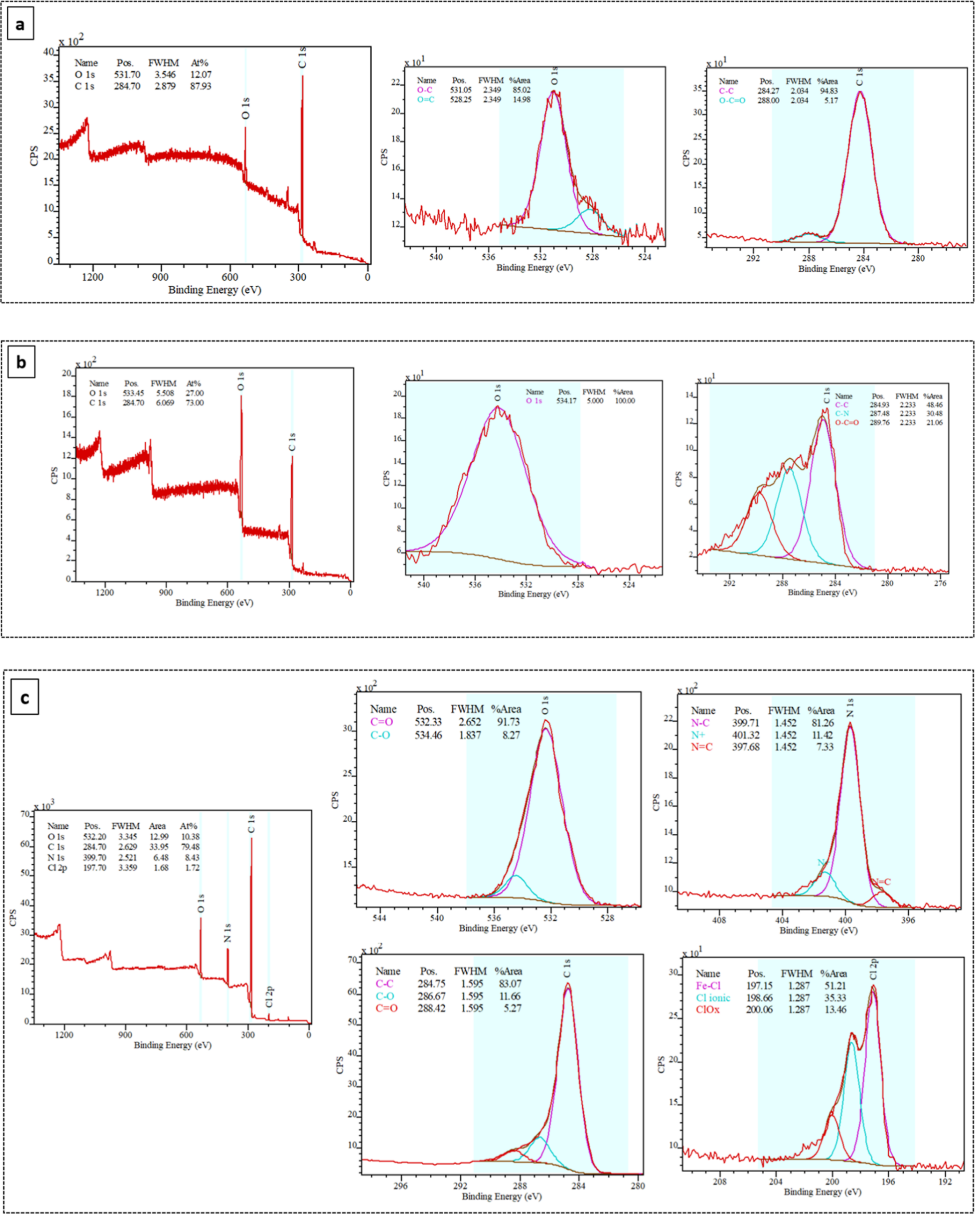
XPS spectrum of: (a) PLGA, (b) PPy-encapsulated, and (c) PPy-coated electrospun mats. The survey, C 1s, O 1s, and N1_S_ spectrum are presented.

### 3.2 Mechanical Properties

It is well-known that the microstructure of the electrospun mats, such as fiber diameter and fiber alignment, directly influences the mechanical behavior of the mats.^41,42^ It is desired that the mats present a mechanical tensile strength close to that of a human autologous nerve (6.5–8.5 MPa).^42^ Moreover, the mats must be flexible enough to be rolled up into a conduit shape. Tensile tests were performed along the fiber axis (0°) to evaluate the mechanical performance of the electrospun mats (Figure 6). All the electrospun mats exhibited tensile strength close to the human autologous nerve, with significant differences from PLGA and PLGA-encapsulated. This increase in tensile strength indicates that the incorporation of PPy nanoparticles reinforced the material within the fibers. No significant difference was found for the Young’s modulus. On the other side, PPy-coated mats showed a decrease in elongation at break. The coating might cause the material to become less flexible.

**Figure 6:**
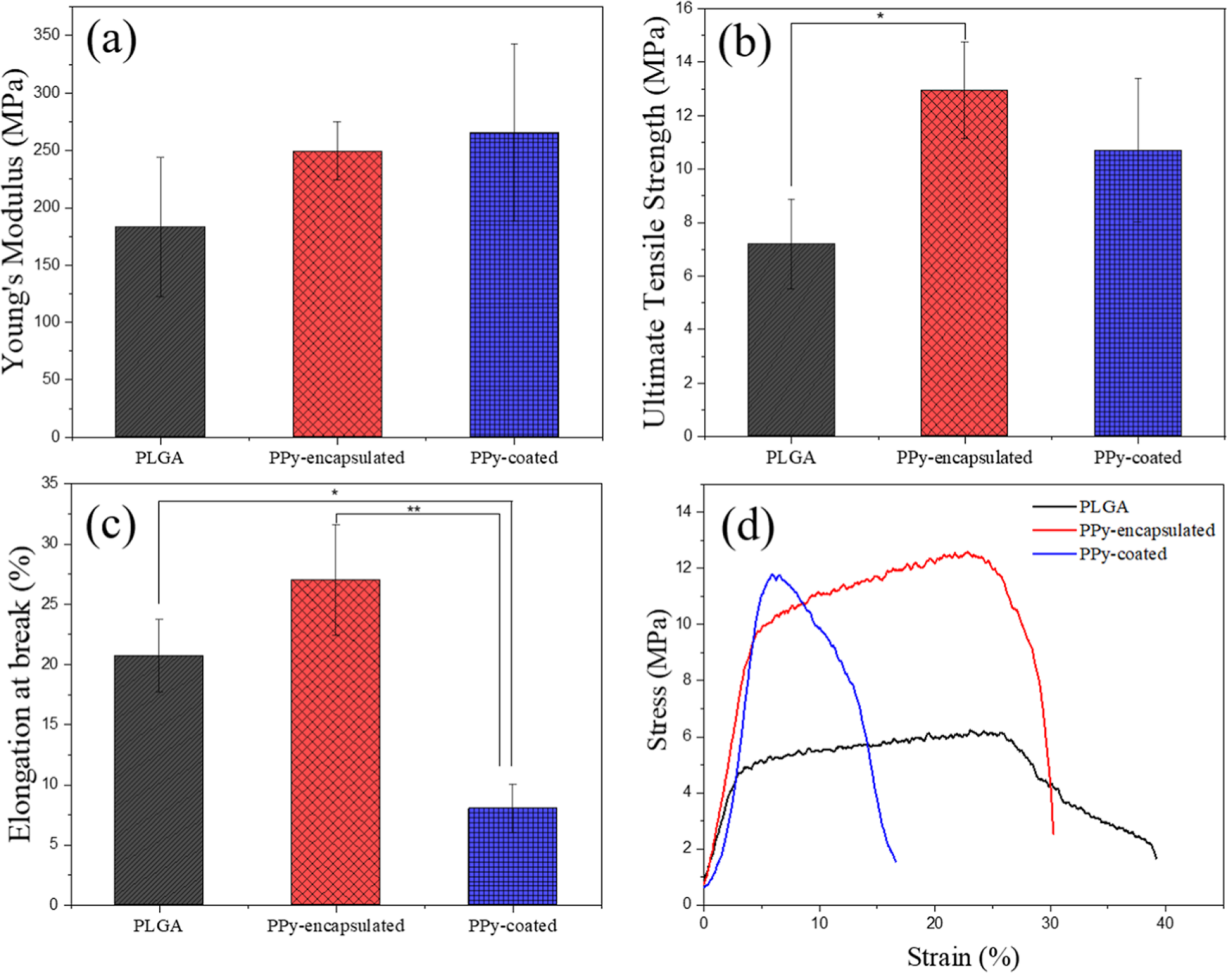
Mechanical properties of PLGA, PPy-encapsulated, and PPy-coated electrospun mats, performed in the longitudinal fiber direction (0°). (a) Young’s modulus, (b) ultimate tensile strength, (c) elongation at break and (d) representative stress x strain curves. *P < 0.05 and **P < 0.01.

Storage modulus curves (*E*′) and loss factor (Tan δ) as a function of the temperature for the PLGA, PPy-encapsulated, and PPy-coated electrospun mats are shown in Figure 7 and Table 1. The *E*′ value at 37 °C was used for this analysis. All the mats demonstrated consistent mechanical mechanical performance, with storage and loss moduli showing thermal stability within the 30–45 °C temperature range, which is essential for in vivo applications. PPy-encapsulated mat presented the highest *E*′ value. This performance can be explained by the mechanical reinforcement provided by PPy nanoparticles in the fibers. Another explanation is that the observed stiffness may also be due to the increased fiber drawing during electrospinning, which may have contributed to the reduced fiber diameter. For the PPy-coated sample, an opposite effect was observed. This effect was expected, due to the low tenacity behavior of PPy, which is on the fiber surfaces. Another piece of information obtained from the curves was the region where the *E*′ is constant. This data is important because it indicates the temperature range over which the material maintains stable mechanical properties. PPy-coated mat kept its mechanical property constant up to 65.5 °C, which is the highest value. This may be due to the presence of the PPy on the surface, which thermally protects the PLGA matrix core. From the loss tangent curves (Tan δ), PPy-coated mats showed a slight shift in *T*_g_ toward to higher temperatures (up to 72 °C), which could be explained by the decrease in the mobility of the PLGA chains due to the presence of the PPy coating on the fiber surfaces.^43^

**Figure 7:**
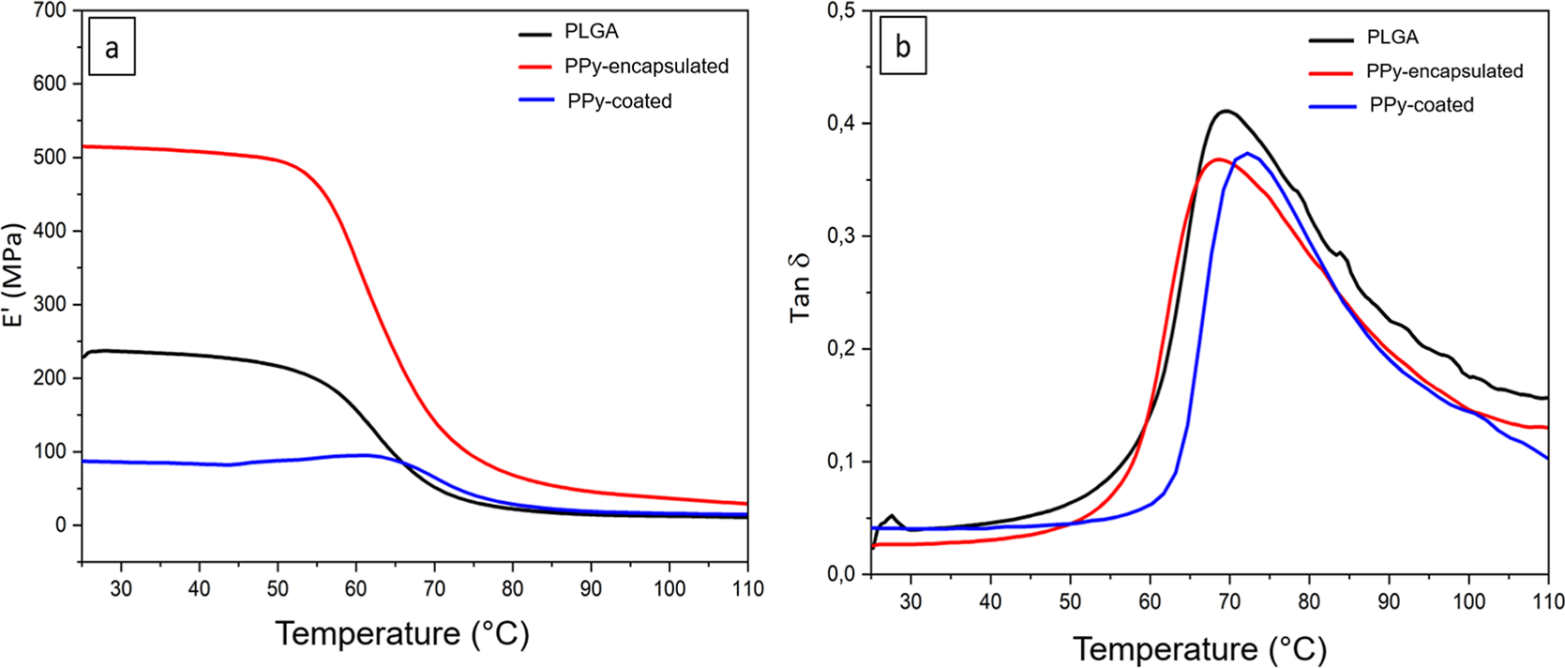
DMA traces (a) storage modulus (*E*′) and (b) loss factor of PLGA, PPy-encapsulated, and PPy-coated electrospun mats.

**Table 1:** DMA Values of Storage Modulus (*E*′) and Loss Factor (Tan δ) of PLGA, PPy-Encapsulated, and PPy-Coated Electrospun Mats

Samples	*E*′ (at 37 °C)	Δ*T E*′ constant (°C)	Tan δ (°C)
PLGA	234.4	25–53.7	69.4
PPy-encapsulated	508.7	25–51.3	68.6
PPy-coated	83.5	25–65.5	72.2

### 3.3 Electrical Properties

The electrical conductivity of PLGA, PPy-encapsulated, and PPy-coated mats is summarized in, Table 2. It can be observed that the electrical conductivity of PPy-encapsulated was not significantly changed by the presence of PPy compared to PLGA mats. This behavior can be explained by microstructure of the mat, which has a fibrous and highly porous structure. The porous structure limits the interaction between the fibers to form conductive pathways. Furthermore, PPy is encapsulated within the fibers, which also prevents the formation of conductive pathways. However, the PPy-coated mats increased the electrical conductivity by 15 orders of magnitude to 10^–2^ S·cm^–1^. It means that the coating was efficient, and the current was conducted along the fibers.

**Table 2:** Electrical Conductivity of PLGA, PPy-Encapsulated, PPy-Coated Electrospun Mats, and PPy Powder

Samples	Electrical conductivity (S·cm^–1^)
PLGA	(2.94 ± 1.43) × 10^–17^
PPy-encapsulated	(5.55 ± 5.44) × 10^–16^
PPy-coated	(5.60 ± 0.03) × 10^–2^
PPy	(1.78 ± 0.06) × 10^–1^

### 3.4 Cytocompatibility Results

The MTT assay is widely performed to assess cytotoxicity by determining cell viability. The results of the MTT assay after 1, 3, and 7 days are shown in Figure 8, with the control group as cells cultured on the well plate, the standard for cell culture. Cells in the control group were considered to have 100% cell viability and the results are expressed as a percentage of the control. According to ISO 10993–5/2009, a metabolic activity above 70% implies that the tested compound has no cytotoxic potential/or has no observable cytotoxicity in the MTT assay.^44^ It was noticed that on day 1 and day 3, PLGA and PPy-encapsulated mats were not cytotoxic. Furthermore, PPy-encapsulated showed slightly higher metabolic activity than neat PLGA mats, showing a high potential to be applied in the human body. However, based on the MTT test results in this study, PPy-coated mats were considered cytotoxic. This cytotoxicity might be associated with residual reaction byproducts from the in situ oxidative polymerization of Py, especially chloride as shown by the XPS results. Another reason could be related to the high Py concentration of 0.05 mol·L^–1^ (50 mM) used in this study. Hence, further studies could be performed with a decreasing Py concentration. Accordingly, Lee et al.^10^ used a Py concentration of 7 mM, which was appropriate to obtain a homogeneous coating on electrospun PLGA fibers, a satisfactory biological response, and an electrical conductivity of 6.72 × 10^–5^ S·cm^–1^. In the study by Song et al. (2016), 10 mM of Py was used for coating PCL electrospun fibers, also with a functional recovery in vivo comparable to nerve autograft.^23^ Nevertheless, the conductivity values presented in these studies were lower compared to the values found in this work, which was 5.60 × 10^–2^ S·cm^–1^. It shows the importance of finding an optimal balance between the electrical conductivity and cytocompatibility of the fibers.

**Figure 8:**
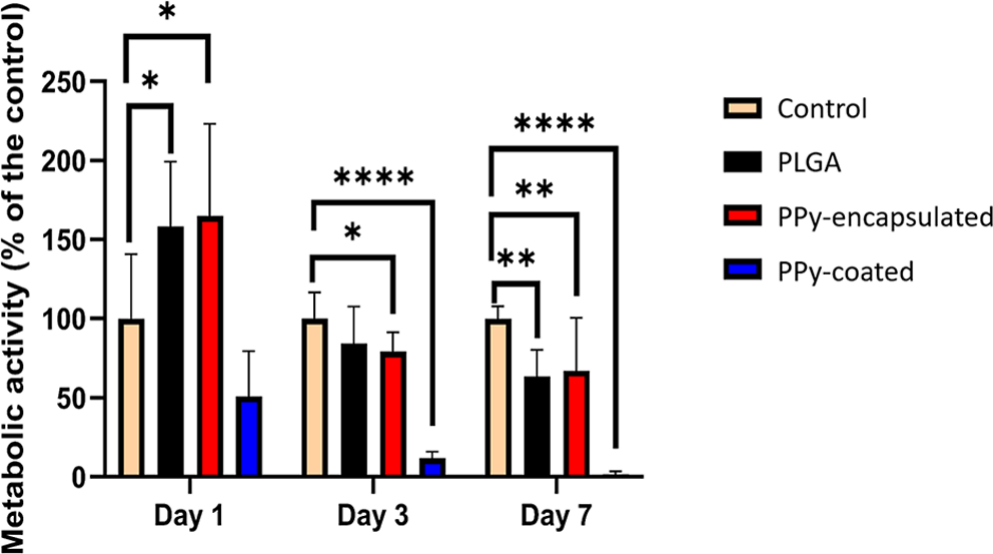
Metabolic activity after 1, 3, and 7 days of the cell viability evaluation of PLGA, PPy-encapsulated, and PPy-coated electrospun mats. Means *P < 0.05, **P < 0.01, ***P < 0.001 and ****P < 0.0001. Results are expressed as mean and standard deviation. Gaussian distribution of the data was analyzed by quantile–quantile (QQ) plot. *N* = 3.

The PC12 cells that were seeded on the electrospun mats are presented in Figure 9. The cells seeded in aligned fibers exhibited a preferential growth direction as shown by the green arrows. As discussed previously, this behavior is beneficial, because it shows that fiber alignment can stretch and guide cells toward their target in vivo applications, supporting tissue regeneration.^45,46^

**Figure 9:**
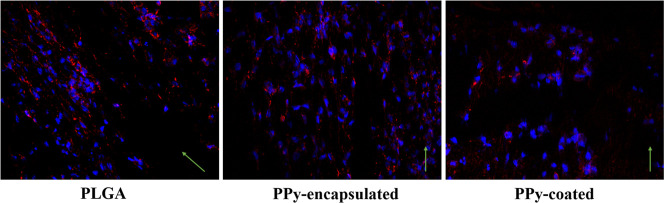
PC12 cells behavior after 7 days on PLGA, PPy-encapsulated, and PPy-coated electrospun mats. The blue staining represents DAPI, which labels cell nuclei, and the red staining corresponds to phalloidin, which shows the actin cytoskeleton.

Prototypes of electrospun conduits are presented in Figure 10. As it can be seen, fiber alignment is in the axial direction of the nerve conduit (Figure 10b). This alignment may promote cell direction and faster cell spreading within the conduit. This feature has a direct impact on faster functional recovery of the nerve, especially in terms of improved motor and sensory functions.^47^ Future in vivo testing is needed to be performed to confirm it. This prototype was developed to analyze challenges of wrapping it up and suturing the conduit prior to surgery, Figure 10. It was observed that both conduits are very sensitive to handling, highlighting the need for a precise and careful surgical approach during handling and surgical procedures. Furthermore, the PPy-coated mats were relatively difficult to roll up due to the loss of flexibility caused by the coating (Figure 10c,d). Since suturing the conduit to close it can be relatively challenging, a surgical glue (e.g., VetbondTM 3 M, USA) could be used as an alternative solution, as reported by dos Santo et al. (2019).^47^ The contrast observed for the resulting electrospun mats from the two route studies showed that future efforts could focus on tuning the fabrication parameters to bridge the gap between materiaĺs properties and biocompatibility. For instance, optimizing the dispersion of PPy within the fibers for the encapsulation route or, testing different parameters for the polymerization of the Py monomer on fiber surface for the coating route.

**Figure 10:**
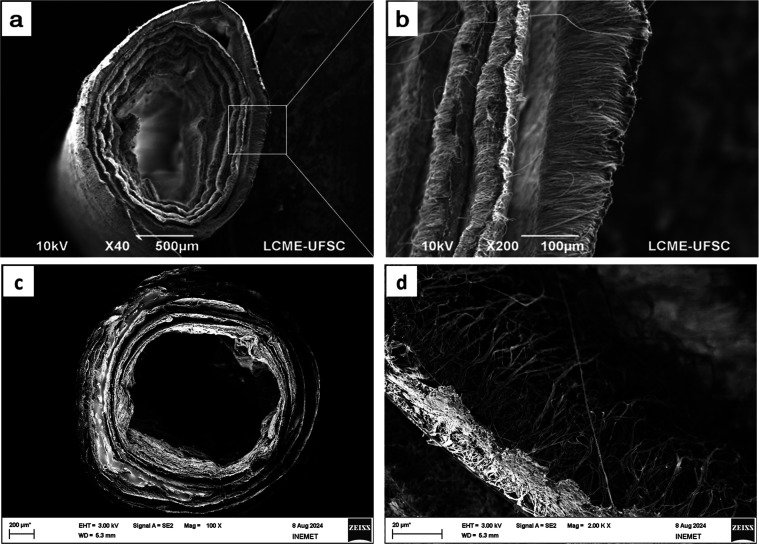
SEM images of nerve conduit, as a prototype of: (a,b) PPy-encapsulated, and (c,d) PPy-coated.

## 4 Conclusion

Aligned conductive electrospun mats were successfully developed by two different processing routes, encapsulation of 7.5 wt % PPy nanoparticles in PLGA fibers and coating PLGA fibers with PPy by in situ oxidative polymerization of Py. Both routes successfully produced aligned mats with bead-free fibers. Encapsulation of the PPy nanoparticles was well achieved, however, agglomerations in some regions were observed. It was shown that the processing routes significantly influenced the properties of the electrospun mats. For instance, it was observed that the PPy-coated mats exhibited higher electrical conductivity and wettability compared to the mats with encapsulated PPy in the PLGA matrix. Although these results were promising, they showed that PPy-coated mats at critical levels presented cytotoxicity according to the results obtained with the MTT test. On the other hand, the encapsulation route showed excellent mechanical performance compared to PPy-coated mats and also compared to nerve autograft data reported in the literature. Additionally, the encapsulation route presented cytocompatibility for up to 7 days. Prototypes of electrospun nerve conduits were successfully developed with the main difficulties being their high mechanical sensibility and flexibility. The PPy-coated mats were unfeasible to roll up due to the loss of flexibility caused by the coating. Other strategies need to be explored to improve handling, which is particularly important during the surgical procedure. The results of this work suggest that the PPy encapsulation route may be preferred for potential nerve tissue applications, as it balances mechanical performance and cytocompatibility, with the potential for further optimization in future research.
