# Correction to “Comparative Analysis of Aligned Conductive Electrospun Mats of PLGA-Polypyrrole for Neural Tissue Engineering: Encapsulation vs Coating”

**DOI:** 10.1021/acsomega.6c02937

**Published:** 2026-07-23

**Authors:** Vanessa Oliveira Castro, Sebastien Livi, Laura Elena Sperling, Marcelo Garrido dos Santos, Patricia Pranke, Katja Heise, Claudia Merlini

**Affiliations:** † Mechanical Engineering Department, Universidade Federal de Santa Catarina (UFSC), 88040-900 Florianópolis, Brazil; ‡ Ingénierie des Matériaux Polymères, Universite Claude Bernard Lyon 1, INSA Lyon, Université Jean Monnet, CNRS UMR 5223, F-69621 Villeurbanne Cédex, France; § Hematology and Stem Cell Laboratory, Faculty of Pharmacy, Universidade Federal do Rio Grande do Sul (UFRGS), 90010-150 Porto Alegre, Rio Grande do Sul, Brazil; ∥ Materials Engineering Special Coordination, Universidade Federal de Santa Catarina (UFSC), 89036-256 Blumenau, Santa Catarina, Brazil; ⊥ Institute of Nanoscale and Biobased Materials, Faculty of Materials Science and Materials Technology, 26545 Technische Universität Bergakademie Freiberg, 09599 Freiberg, Germany

We would like to correct our paper by adding Patricia Pranke, as an author. Dr. Patricia Pranke provided critical expertise in electrospinning and offered ongoing scientific supervision and discussions of the results. The changes are reflected in the authorship of this correction.

